# Glucagon-like Peptide-1 Receptor Agonists and Platelet Function: Potential Benefits Beyond Glycemic Control

**DOI:** 10.3390/ph19030462

**Published:** 2026-03-12

**Authors:** Maria Xanthopoulou, Paschalis Evangelidis, Dimitrios Poulis, Eleni Gavriilaki, Nikolaos Kotsiou, Christina Antza, Vasilios Kotsis, Chrysoula Doxani, Theodoros Mprotsis, Elias Zintzaras, Panagiota Anyfanti

**Affiliations:** 1 Third Department of Internal Medicine, Papageorgiou Hospital, Aristotle University of Thessaloniki, 56429 Thessaloniki, Greece; xanthopouloum6@gmail.com (M.X.); kris-antza@hotmail.com (C.A.); vkotsis@auth.gr (V.K.); panyfanti@auth.gr (P.A.); 2 Second Propedeutic Department of Internal Medicine, Hippokration Hospital, Aristotle University of Thessaloniki, 54642 Thessaloniki, Greece; elenicelli@yahoo.gr (E.G.); kotsiounikolaos@gmail.com (N.K.); 3 Department of Biomathematics, Faculty of Medicine, University of Thessaly, 41222 Larissa, Greece; doxani@uth.gr (C.D.); tmprotsis@uth.gr (T.M.); zintza@uth.gr (E.Z.); 4 Center for Clinical Evidence Synthesis, The Institute for Clinical Research and Health Policy Studies, Tufts Medical Center, Tufts University School of Medicine, Boston, MA 02111, USA; 5 Pharmacology and Drug Development Program, Sackler School of Graduate Biomedical Sciences, Tufts University School of Medicine, Boston, MA 02111, USA

**Keywords:** atherothrombosis, cardiovascular disease, endothelium, GLP-1 receptor agonists, nitric oxide, platelet activation, platelet aggregation, thrombosis, thromboxane

## Abstract

There is cumulative evidence that glucagon-like peptide-1 receptor agonists (GLP-1 RAs) can offer cardiovascular protection extending beyond glucose-lowering and weight reduction, but the underlying mechanisms contributing to these effects remain incompletely understood. Modulation of platelet function might contribute to the aforementioned benefits. In the current literature review article, we synthesized available preclinical and clinical data evaluating the effects of GLP-1 RAs on platelet activation and function. Preclinical data indicate that GLP-1 RAs might decrease platelet activation via both GLP-1 receptor-dependent and -independent mechanisms with the involvement of cyclic adenosine monophosphate signaling, increase in nitric oxide bioavailability, and suppression of thromboxane-mediated pathways, particularly under inflammatory or shear-stress conditions. Additionally, clinical studies, despite being limited and heterogeneous, support a reduction in platelet activation markers, even independently of glycemic control or weight loss. However, most of them are characterized by small sample sizes and significant heterogeneity among them. In summary, existing evidence suggests that GLP-1 RAs exhibit potential antiplatelet effects that could contribute to their cardioprotective profile. Larger, well-designed clinical studies are crucial to better understand the clinical importance of platelet modulation by GLP-1 RAs and their potential implications for cardiovascular risk reduction.

## 1. Introduction

Incretins are a class of gut-derived peptide hormones that stimulate insulin release in response to nutrient ingestion in a glucose-dependent manner, thereby acting as major regulators of postprandial glucose regulation. Glucose-dependent insulinotropic polypeptide and glucagon-like peptide-1 (GLP-1) are the two primary incretins identified in humans, both acting through specific G protein-coupled receptors predominantly expressed on pancreatic β-cells [1]. Pharmacological exploitation of the incretin system has led to the development of GLP-1 receptor (GLP-1R) agonists (GLP-1 RAs), with rapidly and continuously increasing prescription patterns despite socioeconomic limitations. Following the Food and Drug Administration’s (FDA) approval of exenatide in 2004 [2,3], GLP-1 RAs were initially introduced as hypoglycemic drugs for the management of type 2 diabetes mellitus (T2DM) and have been further established as foundational therapies in the management of obesity regardless of the presence of diabetes [3]. The first oral GLP-1 RA, semaglutide, is now broadly available in the US following recent FDA approval for weight loss in adults and is expected to substantially expand the GLP-1 RA market in the near future [4].

However, the beneficial effects of treatment with GLP-RAs expand far beyond their efficacy on glucose and weight control. Treatment with GLP-1 RAs improves several parameters of metabolic syndrome, including blood pressure and nonalcoholic fatty liver disease, but most importantly, it is associated with improved cardiovascular outcomes [5]. As a result, GLP-1 RAs have been introduced as a first-line medication for patients with diabetes and prior cardiovascular events according to the American Diabetes Association guidelines on pharmacotherapy issued in 2025 [6]. As the spectrum of diseases for which GLP-1 RAs may be used is continuously expanding from cardiovascular and liver disease to neurodegenerative disease and substance abuse disorders, multiple studies have been conducted to elucidate their physiological role in different systems besides the enhancement of insulin secretion, early satiety, and the delay in gastric emptying [7]. Nevertheless, the effects of GLP-1 RAs on the vascular system remain largely unexplored.

Subclinical vascular injury is considered an early event in the course of cardiovascular diseases, triggered by primary pathophysiological processes including endothelial dysfunction and thromboinflammation, which are mechanistically linked to cardiovascular disease [8]. Platelets bridge inflammation and thrombosis and play a pivotal role in the development of atherothrombosis. Platelets not only form thrombi subsequent to plaque rupture but also act as inflammatory effectors contributing to early atherogenesis through a constant cross-talk with other inflammatory mediators, promoting leukocyte recruitment and chronic inflammatory changes within the vascular wall [9]. Platelet activation is evident in obesity, prediabetes, and diabetes, thereby supporting early atherogenic processes in the context of these diseases [10,11,12]. Considering the favorable effects of GLP-1 RAs on the cardiovascular system, it could be hypothesized that these could be at least partially mediated through modulation of platelet function or activity.

Although the cardiovascular benefits of GLP-1 RAs have been extensively reviewed, a focused synthesis of their effects on platelet function is currently lacking. Therefore, we decided to organize the present narrative review article to summarize available data on the potential effects of treatment with GLP-1 RAs on platelet function. A brief overview of preclinical research will be provided, and special emphasis will be placed on data derived from studies conducted in humans. Specifically, in this narrative literature review, a synthesis of the available preclinical and clinical data in this field is provided. Given its non-systematic design, indirect mechanistic evidence, such as endothelial function, inflammatory pathways, and nitric oxide signaling, is presented to elucidate potential platelet-related effects, while acknowledging the heterogeneity and the potential limitations of experimental studies.

## 2. Effects of Treatment with GLP-1 RAs on Atherothrombotic Events: Summary of Data from Randomized Controlled Trials (RCTs)

The cardio-protective effects of treatment with GLP-1 RAs have been overtly demonstrated in multiple randomized controlled trials (RCTs) assessing hard cardiovascular endpoints among individuals with T2DM, atherosclerotic cardiovascular disease, or high cardiovascular risk, as summarized in detail elsewhere [13]. A reduction in major adverse cardiovascular events (MACE) appears to be a class effect of GLP-1 RAs, although the magnitude, consistency, and spectrum of benefit may differ among different agents. More specifically, a decade ago, liraglutide was found to reduce the rate of occurrence of cardiovascular death, myocardial infarction, and non-fatal stroke in a time-to-event analysis by the LEADER investigators when added to standard care in high cardiovascular risk type-2 diabetes mellitus (T2DM) patients [14]. Similar outcomes were confirmed in a sub-analysis of the DEVOTE-10 trial, a multi-center double-blind, randomized trial, where 7637 patients with T2DM at high cardiovascular risk receiving at least one oral or injectable GLP-1RA were randomized to degludec or glargine in order to assess cardiovascular safety [15]. The Harmony trial found that albiglutide was superior to placebo regarding the first occurrence of the aforementioned MACE in T2DM patients with previously established cardiovascular, cerebrovascular, or peripheral artery disease [16]. In the REWIND clinical trial, the addition of weekly dulaglutide to standard treatment was found to reduce the occurrence of stroke, myocardial infarction, or cardiovascular death in T2DM individuals, among whom 31.5% had previously established cardiovascular disease. This effect was consistently independent of prior MACE, implying that dulaglutide use could be considered for primary as well as secondary prevention of cardiovascular disease [17].

Interestingly, semaglutide reduced the incidence of MACE in obese individuals with established cardiovascular disease in the SELECT trial for semaglutide versus placebo, irrespective of initial or post-treatment levels of hemoglobin A1c (HbA1c), which ranged from normal to prediabetic levels. A multifaceted impact on atherogenesis was deduced from the study, based on the observed reduction in C-reactive protein (CRP) and improvement in renal function associated with semaglutide use [18]. The effects of semaglutide on T2DM patients receiving standard care on the incidence of MACE were further assessed in SUSTAIN-6, which showed a 26% risk reduction driven by a significant reduction in the incidence of non-fatal stroke and a non-significant reduction in myocardial infarction and cardiovascular death [19]. The subcutaneous route of administration is a main concern for the widespread use and long-term adherence to GLP-1 RAs [20]. The SOUL trial compared the effects of oral semaglutide versus placebo in a large group of TD2M patients who were stratified according to baseline or subsequent sodium-glucose cotransporter 2 inhibitor use and showed a 14% risk reduction for MACE in the intervention arm [21].

Collectively, there is sound evidence from large RCTs that treatment with GLP-1 RAs results in significant improvements in hard cardiovascular endpoints. This effect is apparently modulated by their impact on glucose control and body weight reduction but also through their pleiotropic effects on a range of cardiometabolic parameters, from blood pressure and lipid profile to renal function and chronic, low-grade inflammation [19,21,22].

## 3. Cardioprotective Mechanisms of GLP-1 RA Treatment: Beyond Glycemic Control and Weight Reduction

The cardioprotective mechanisms of GLP-1 RAs have evolved into a fascinating area of research in the cardiovascular field. There is evidence from preclinical and clinical studies that GLP-1 RAs act on pathophysiological pathways that are critical to the development and progression of atherothrombosis. These agents have been found to have multifaceted effects on endothelial function [23]. Flow-mediated dilation (FMD), the gold-standard vascular measure of endothelial dysfunction, was increased while carotid-femoral pulse wave velocity was diminished, more importantly with liraglutide versus metformin in a group of 60 newly diagnosed T2DM patients [24]. This effect on FMD was consistently described in multiple trials that assessed pre- and post-treatment endothelial function [24,25,26].

In line with the above, a 12-week exenatide treatment versus placebo was associated with a significant reduction in circulating serum adhesion molecules such as soluble intercellular adhesion molecule-1 and vascular cell adhesion molecule-1, along with a significant increase in coronary flow velocity reserve, which serves as an indicator of coronary endothelial dysfunction [27]. In vitro studies performed by the same researchers found that incubation of human umbilical vein endothelial cells with exendin-4, a form of exenatide, induced a dose-dependent increase in nitric oxide (NO) production (whose loss of bioavailability is a key component of atherosclerosis) and in phosphorylation of endothelial NO synthase (eNOS) [27]. In addition to its overall positive effect on the metabolic syndrome biochemical indices confirmed by the in vivo study [waist circumference, fasting plasma glucose (FPG), triglycerides, and low-density lipoprotein (LDL) cholesterol reduction, high-density lipoprotein cholesterol increase, marginal reduction in blood pressure], exendin-4 was found to protect against homocysteine-induced coronary artery endothelial impairment in the same study [27].

Other widely established markers of the atherosclerotic process have been shown to be positively affected by treatment with GLP-1 RAs. Carotid intima media thickness was normalized when added to metformin treatment in incretin-naive T2DM patients in an 18-month prospective study performed by Rizzo et al. [28]. In addition, GLP-1 treatment reduces oxidative stress and inflammation, an effect that has been underscored by a marked reduction in oxidized malondialdehyde and protein carbonyls [24], nitrotyrosine, high-sensitivity C-reactive protein (hs-CRP), and interleukin (IL)-6 [25]. GLP-1 RAs appear to modify the role of key molecules participating in the atherosclerotic process, such as monocyte chemoattractant protein-1 (MCP-1), which modulates macrophage attraction within the oxidized LDL-containing endothelium. MCP-1 was found to be significantly decreased in patients receiving exenatide in a clinical trial comparing the effects of exenatide versus insulin glargine on several biomarkers in insulin and incretin-naive individuals receiving metformin-only treatment [26]. In line with this finding, Hachula et al. demonstrated a reduction in MCP-1 and L-selectin with GLP-1 RA treatment in diabetic patients with atherosclerosis [29].

The beneficial effects of GLP-1 RA treatment on vascular inflammation and endothelium have been further verified by in vivo studies. A sub-study of the LIRAFLAME double-blind RCT included 30 individuals who underwent [^64^Cu]Cu-DOTATATE positron emission tomography/computed tomography (PET/CT) and computed tomography (CT) for calculation of coronary artery calcium score at baseline and at the end of a 26-week treatment with either liraglutide or placebo, with assessment of coronary artery inflammation as the primary endpoint, as reflected by the SUVmax (maximum standardized uptake value) and mean SUVmax change from baseline until the end of treatment. Results showed a significant decrease in the liraglutide group for SUVmax, thus indicating lower inflammatory burden, in addition to a weak positive correlation between [^64^Cu]Cu-DOTATATE uptake at baseline and hs-CRP levels [30]. In addition, 6-month treatment with liraglutide resulted in a greater improvement in endothelial function as assessed with flow-mediated dilation, arterial stiffness, left ventricular myocardial strain, twisting and untwisting, N-terminal pro–B-type natriuretic peptide, and oxidative stress than metformin in newly diagnosed and treatment-naive patients with T2DM [24].

Although GLP-1 RAs appear to have a clear impact on endothelial dysfunction, vascular inflammation, and oxidative stress, their impact on platelet function remains less well defined in the available literature. Considering the incremental role of platelet activation in the pathogenesis of atherothrombosis, a thorough understanding of the potential effects of GLP-1 RAs on platelet function is critical to elucidate the mechanisms underlying the cardioprotective benefits associated with treatment. The potential for modulating platelet function and signaling by GLP-RAs could affect therapeutic decisions for cardiovascular disease prevention and treatment in high-risk individuals, thereby emerging as an important research hypothesis.

## 4. GLP-1 RAs and Platelet Function: Preclinical Data

There is emerging evidence arising mainly from preclinical and experimental studies that GLP-1 RAs reduce platelet activation and aggregation. Relevant studies are summarized in Table 1. Cameron-Vendrig and colleagues were among the first to address this hypothesis by examining the impact of a GLP1-RA (exenatide) on human platelets in an in vitro study [31]. Both endogenous GLP-1 and exenatide significantly delayed and reduced thrombin-induced platelet aggregation (GLP-1 at 10^−9^ M produced a 60% reduction at 1 min). Aiming to elucidate the underlying mechanism, GLP-1R mRNA and cyclic adenosine monophosphate (cAMP) levels were measured in a megakaryocyte cell line. Specifically, low but measurable GLP-1R mRNA expression and a small cAMP increase were noticed after incubation with GLP-1. Remarkably, an increase in intracellular cAMP and protein kinase A (PKA) activation is crucial for suppression of platelet activation [32]. The same research group, in a subsequent work, confirmed the expression of GLP-1R in a human megakaryocyte line, whereas incubation with endogenous GLP-1 or exenatide induced cAMP response [33]. Moreover, human platelets and mouse whole blood were incubated with the GLP-1RA, and a significant suppression of thrombin, adenosine diphosphate (ADP), and collagen-induced aggregation was reported. In both normoglycemic and hyperglycemic mice, administration of exenatide reduced thrombus formation. Importantly, when the mice received bone marrow lacking GLP-1R (GLP-1R-/-), the antithrombotic effect was attenuated, while in those also missing the eNOS gene, the outcome was undetectable, suggesting a potential involvement of NO signaling as well.

Indeed, there are studies supporting the hypothesis that GLP-1RA-mediated reduction in platelet activation might be NO increase-dependent [34], autonomous from GLP-1R pathways. In the study of Barale et al., platelets were isolated from 72 healthy individuals and incubated with GLP-1 endogenous peptide, a GLP-1 metabolite, and liraglutide [34]. Interestingly, platelet aggregation in response to collagen and arachidonic acid was not reduced by these molecules. Nevertheless, when platelets were pre-treated with a NO donor (sodium nitroprusside), the administration of any of these GLP-1-associated peptides significantly enhanced the NO-mediated anti-aggregatory action. At the molecular level, levels of which amplify NO expression were increased, along with enhanced vasodilator-stimulated phosphoprotein phosphorylation and reduced reactive oxygen species production. These effects did not require the presence of a classical GLP-1 R. Therefore, GLP-1-related peptides might improve the antithrombotic and antiplatelet effects of NO under conditions where NO is present or of endothelial activation. Moreover, independently, they do not exhibit a strong inhibitory effect on platelet activation in resting conditions.

Sternkopf and colleagues have utilized ex vivo microfluidic whole blood assays under shear flow and animal models to investigate the effects of both endogenous, intact GLP-1 (7–36) and GLP-1 (9–36), a form derived by dipeptidyl peptidase 4 (DPP-4) cleavage [35]. The authors reported that intact GLP-1 significantly reduced the volume of thrombus in human whole blood under flow, whereas GLP-1 (9–36) did not. Additionally, in mice, pharmacologic inhibition and genetic deletion of DPP-4 suppressed flow-dependent platelet aggregation, but this effect was not identified in GLP-1R gene knockout (GLP1R-/-) animals. Thus, it can be speculated that GLP-1-mediated platelet aggregation inhibition is GLP-1R-independent and that native GLP-1 acts as a suppressor of thrombus growth under physiological conditions.

Interestingly, GLP-1 RAs and DPP-4 inhibitors have been found to suppress platelet activation, microvascular thrombosis, oxidative stress, and endothelial dysfunction in the sepsis mouse model [36]. Specifically, this effect may be mediated via a cAMP/PKA-dependent signaling pathway in platelets and monocytes, whereas it was previously described as GLP-1R-dependent, contrary to the current study [36]. Similarly, liraglutide has been proposed to decrease platelet recruitment to the lung tissue of mice with aspirin-exacerbated respiratory disease (AERD) [37]. Furthermore, in this study, platelets from AERD patients and healthy controls were also stimulated in vitro with a thromboxane receptor agonist and pre-treated with liraglutide: those platelets displayed reduced activation, indicated by lower CD62P (i.e., P-selectin) expression and release of chemokine C-X-C motif ligand 7, a molecule secreted by activated platelets. Nevertheless, in a recently presented abstract at the Joint Irish-United Kingdom Endocrine Meeting 2024, liraglutide (20 µM), dulaglutide (20 µM), and danuglipron (30 µM) were not found to exhibit antiplatelet activation and aggregation properties [38] under basal conditions. However, the small sample size (*n* = 3–5) and the high concentrations of the drugs used during incubation are important limitations to the study findings. These findings are consistent with other in vitro studies showing minimal or even absent antiplatelet effects of GLP-1 RAs under resting conditions, suggesting that the inhibitory effects might be context-dependent and more prominent in inflammatory or shear stress models, as also discussed below.

**Figure 1:**
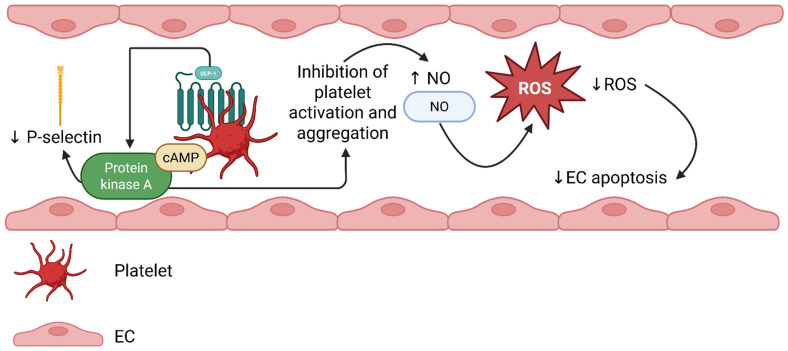
Proposed mechanisms by which GLP-1 RAs might modulate platelet activation and thrombus formation. Receptor-dependent pathways (GLP-1R/cAMP/protein kinase signaling) and receptor-independent mechanisms (NO–cGMP signaling and oxidative stress modulation) are presented separately for conceptual clarity. Created in BioRender. Evangelidis, P. (2025) https://BioRender.com/qkhovao (accessed on 25 November 2025). cAMP: cyclic adenosine monophosphate, EC: endothelial cell, GLP-1 RA: glucagon-like peptide-1 receptor agonists, GLP-1: glucagon-like peptide-1, NO: nitric oxide, ROS: reactive oxygen species, ↓: decrease, ↑: increase.

Overall, preclinical studies suggest that GLP-1-RAs can reduce platelet activation via two potential pathways: a direct, GLP-1R-dependent pathway, with the involvement of the cAMP/PKA pathway, implicated mainly under inflammatory or thromboxane-mediated conditions, and a GLP-1R-independent one. In the last one, GLP-1-associated peptides might activate NO-cGMP signaling, suppressing oxidative stress, but only when NO or shear stress is present [39]. Moreover, native GLP-1 molecules seem to function as shear-dependent suppressors of thrombus formation, while classical GLP-1R agonists do not consistently inhibit platelet activation under basal conditions in healthy platelets, as has been reported from in vitro studies. Therefore, GLP-1RA antiplatelet effects might be more profound under a state of inflammation, endothelial activation, or physiological flow. The proposed mechanism is described in Figure 1.

It should be highlighted that the biological effects of native GLP-1 peptides, GLP-1 metabolites, and pharmacological GLP-1 RAs are not necessarily interchangeable. Differences in receptor dependency, signaling pathway activation, and methodology in experimental studies might explain the heterogeneity of findings reported in the aforementioned studies. Therefore, caution is needed when interpreting platelet-related effects across different GLP-1–based molecules. Moreover, expression of GLP-1R on platelets is not universally accepted, and discrepancies across the above-described studies may partly reflect methodological differences, including the use of flow cytometry versus transcriptomic approaches, variability in antibody specificity, and differences in platelet activation status at the time of evaluations. Importantly, detection of GLP-1R mRNA transcripts does not necessarily equate to the presence of a functional receptor at the protein level capable of mediating intracellular signaling, which may partly explain discordant functional results across studies. Consequently, receptor-independent mechanisms may be equally or even more relevant in certain experimental settings.

**Table 1:** Preclinical studies examining the impact of GLP-1 RAs on platelet function.

Reference	GLP-1 Peptide/RA	Experimental Model	Study Type	Platelet Stimulus	Assays	Main Findings
Cameron-Vendrig et al., 2012 [31]	Endogenous GLP-1 and GLP-1 RA (exenatide)	Freshly isolated gel-filtered human platelets in normoglycemic conditions	In vitro human platelet study	Thrombin	Light-transmission aggregometry Intracellular cAMP Western blot/RT-PCR for GLP-1R detection in platelets/megakaryocyte cell line	Pre-incubation with GLP-1 or exenatide for 15 min significantly delayed ↓ thrombin-induced platelet aggregation ↓ expression of GLP-1 mRNA on platelets/megakaryocytes
Cameron-Vendrig et al., 2016 [33]	Endogenous GLP-1 and GLP-1 RA (exenatide)	Human platelets-MEG-01 cell lines (in vitro) Mouse artery laser injury model (in vivo)	Mixed in vitro and in vivo	Aggregation: thrombin, ADP, and collagen Thrombus formation: artery laser injury	Light transmission aggregometry Whole blood perfusion under flow cAMP response in MEG-01 cells Intravital microscopy of thrombus formation	Exenatide: significantly inhibited platelet aggregation induced by thrombin, ADP, and collagen, ↓ thrombus formation under flow, and in a mouse model Thrombus formation was ↑ in mice treated with GLP1R-/- BM compared to those with WT
Barale et al., 2017 [34]	Endogenous GLP-1 [GLP-1 (7–36)], GLP-1 metabolite [GLP-1 (9–36)] and GLP-1 RA (liraglutide)	PRP from healthy volunteers	In vitro human platelet study	Collagen, arachidonic acid, SNP	Light transmission aggregometry PFA-100 cGMP, cAMP, VASP phosphorylation, GLP-1R expression (flow-cytometry), ROS production	Platelets express GLP-1R (mean: 71.5 ± 1.8%) GLP-1 (7–36), GLP-1 (9–36), and liraglutide alone did not significantly ↓ aggregation ↑ NO donor (SNP): GLP-1 RA anti-aggregatory effect cGMP production in response to SNP ↑ by GLP-1 peptides, VASP-phosphorylation ↑, and ROS production ↓
Steven et al., 2017 [36]	GLP-1 RA (liraglutide) and DPP-4 inhibitor (linagliptin)	Human platelets and monocytes (in vitro) LPS-induced endotoxemia in WT, DPP-4 knockout, and GLP-1R knockout mice (in vivo)	Mixed in vitro and in vivo	LPS	cAMP, PKA Platelet activation GLP-1R in platelets Imaging for microvascular thrombosis in the lungs Platelet counts and LDH ROS markers	GLP-1R activation or DPP-4 inhibition ↓ LPS-induced thrombocytopenia, microvascular thrombosis, endothelial dysfunction, oxidative stress, and ↑ survival in mice In human platelet assays, GLP-1 RA inhibited platelet activation and ↑ cAMP in a GLP-1R-dependent manner
Sternkopf et al., 2020 [35]	Native intact GLP-1 (7–36), DPP-4-cleaved GLP-1 (9–36)	Human whole blood under shear-flow, mouse blood	Mixed in vitro and ex vivo	Collagen-dependent adhesion, thrombus formation under shear flow	Microfluidic assays of thrombus volume under flow In vivo platelet aggregation in mice with DPP-4 inhibition/knockout and GLP-1R knockout GLP-1R transcripts in human platelets	Native GLP-1 (7–36) ↓ thrombus volume under both venous and arterial shear DPP-4 inhibition/deficiency ↓ flow-dependent platelet aggregation in mice, while GLP-1R knockout did not ↓ this effect, suggesting a GLP-1R-independent pathway
Foer et al., 2023 [37]	GLP-1 RA (liraglutide)	Human: platelets from patients with AERD and healthy controls, stimulated in vitro with a TXA RA Murine: AERD-like mouse model, treated with liraglutide	Mixed human ex vivo, murine in vivo, and in vitro platelet activation	TXA RA (for human platelets), lysin-aspirin challenge in mice	GLP-1R expression (flow-cytometry) Platelet activation markers (CD62P expression) and CXCL7 release in human platelets	Human platelets exposed to liraglutide showed ↓ TXA-induced activation (↓ CD62P, ↓ CXCL7 release) In mice, a single dose of liraglutide ↓ lysin-aspirin induced airway resistance and ↓ platelet recruitment to lung tissue
Kumar et al., 2024 [38]	GLP-1 RAs: liraglutide, dulaglutide, danuglipron	PRP from healthy volunteers	In vitro human platelet study	ADP, arachidonic acid, epinephrine, collagen	Light transmission aggregometry CD62P, PAC-1 for platelet activation (flow-cytometry)	No significant difference in platelet aggregation or activation marker expression (CD62P, PAC-1) after in vitro incubation with any of the three GLP-1R agonists in healthy donor platelets

Note: Considerable heterogeneity exists among preclinical studies with respect to experimental models, platelet stimuli, drug concentrations, and methodological approaches. AA: arachidonic acid, AERD: aspirin-exacerbated respiratory disease, ADP: adenosine diphosphate, BM: bone marrow, cAMP: cyclic adenosine monophosphate, cGMP: cyclic guanosine monophosphate, CXCL7: chemokine (C-X-C motif) ligand 7, DPP-4/DPP-4: dipeptidyl peptidase-4, eNOS: endothelial nitric oxide synthase, GLP-1: glucagon-like peptide-1, GLP-1R: glucagon-like peptide-1 receptor, GLP-1 RA: glucagon-like peptide-1 receptor agonist, KO: knockout, LDH: lactate dehydrogenase, LPS: lipopolysaccharide, MEG-01: human megakaryocyte cell line 01, NO: nitric oxide, PKA: protein kinase A, PFA-100: platelet function analyzer-100, PRP: platelet-rich plasma, ROS: reactive oxygen species, RT-PCR: reverse transcription polymerase chain reaction, SNP: sodium nitroprusside, TXA RA: thromboxane receptor agonist, VASP: vasodilator-stimulated phosphoprotein, WT: wild-type, ↓: decrease, ↑: increase.

## 5. Effects of GLP-1 RAs on Platelet Function: Clinical Studies

Clinical studies on human subjects that explored the effect of GLP-1 treatment on platelet function markers are summarized in Table 2. The single-center case–control study conducted by Çalapkulu et al. investigated the effects of a six-month treatment of exenatide on T2DM patients on platelet indices [platelet count, platelet distribution width (PDW), mean platelet volume (MPV)], and glucose tolerance markers [HbA1c, FPG, postprandial glucose, and body mass index (BMI)], while also exploring the correlation between them [40]. A positive correlation between both PDW (an indicator of heterogeneity of platelet size which is elevated in the presence of activated platelets) and MPV (indicative of the presence of large platelets which are more active hemostatically) and BMI, FPG, HbA1c, along with a statistically significant decrease in platelet levels and PDW, was observed after completion of the treatment period, while MPV level was not diminished significantly. No correlation was observed between the reduction in PDW or platelet count and either BMI or HbA1c decrease. Hence, the authors have suggested that the effect of exenatide on platelet function is independent of hyperglycemia or BMI reduction [40].

The interventional study carried out by Kahal et al. explored the effect of liraglutide on platelet function in women with polycystic ovary syndrome (PCOS) and healthy controls using flow cytometry and measurements of fibrinogen binding and P-selectin expression, as well as platelet response to activation by ADP or prostaglandin (PG)I2 [41]. P-selectin levels were found to have significantly decreased with treatment in the control group as opposed to the PCOS group, while levels of fibrinogen were unchanged in both groups [41]. The different rates in platelet function response to liraglutide treatment between the two groups have been attributed to a probable inherent defect in platelet reactivity for women with PCOS, which can partly be explained by reduced sensitivity of platelets in states of insulin resistance, such as diabetes mellitus, obesity, and PCOS, to the anti-aggregating effects of insulin. This phenomenon has been described in an in vitro study conducted by Trovati et al., who investigated the platelet aggregation responses in platelet-rich plasma incubated with insulin from obese diabetic individuals versus healthy donors [42]. These results also suggest that reduced platelet activation indices—in this case, P-selectin—are associated with body weight reduction.

The ELAID pilot study investigated the effects of liraglutide treatment versus placebo on platelet aggregation under the influence of different agonists, namely arachidonic acid, ADP, epinephrine, ristocetin, and saline 0.9%, in subjects with T2DM without established macrovascular complications and not receiving antiplatelet treatment. Normal aggregation was identified in both groups, whereas a transient attenuation of the maximum slope of aggregation response was identified between days 0 and 7 for the liraglutide group in the presence of certain agonists. This attenuation of the max slope has been postulated to be either GLP-1R-related or related to NO-generated mechanisms. As denoted by the authors, these outcomes need to be reproduced in larger trials with regard to their biological significance [43].

The parallel arm RCT conducted by Simeone et al. demonstrated the effects of a 7% weight loss attained by either a GLP1-RA or lifestyle changes in patients with impaired glucose tolerance or impaired fasting glucose or T2DM on platelet activation and lipid peroxidation, as reflected by the urinary excretion of urinary 11-dehydro-thromboxane (TX)B2 and urinary 8-iso-PGF2a, which were both found to have decreased with either intervention; these metabolites had been linked to android obesity-related and diabetes mellitus-related platelet activation by Davi et al. and Santilly et al., respectively [10,11]. As denoted by the authors, the trial design aimed to assess the thromboxane-related platelet activation, which is evident even at early stages of diabetes mellitus, and to dissect the relative contribution to liraglutide, per se, or weight loss on platelet activation markers to conclude that an independent of the concomitant weight loss mechanism of action of liraglutide on platelet aggregation cannot be excluded [44].

Furthermore, Zhang et al. conducted a controlled trial involving thirty T2DM patients, who received exenatide for eight weeks, and thirty controls to investigate the effects on the aforementioned intervention on coagulation and platelet activation; markers of platelet activation P-selectin and PAC-1 (i.e., activated glycoprotein IIb/IIIa) and platelet aggregation rates in patients with T2DM were lowered after treatment, while the difference (δ-) in platelet aggregation rates were positively correlated with most parameters including δ BMI, δ P-selectin, δ waist circumference, δ FPG, and δ HbA1C and negatively correlated with δ NO. These outcomes are in line with previous data, which suggest that GLP1-RA treatment reduces platelet activation through an increase in NO bioavailability, along with modulation of dyslipidemia and hyperglycemia, both of which are associated with altered platelet function [45].

Finally, the interventional trial conducted by Cahill et al. enrolled individuals with obesity and prediabetes. After establishing GLP-1R expression on platelets in whole blood specimens and GLP-1R-related GLP-1RA action in platelet-rich plasma, patients were randomized in a 2:1:1 ratio to receive liraglutide or sitagliptin, or caloric restriction alone, and platelet-rich plasma samples were stimulated with U44619, a PGH2-stable analog, to assess thromboxane-induced platelet aggregation at baseline and post-treatment. Investigators found that exposure to liraglutide significantly reduced TX-induced platelet aggregation in comparison to weight loss or sitagliptin treatment, further supporting a direct effect of GLP-1R-dependent action of GLP-1 RAs on platelets [46].

Overall, the assumed mechanisms resulting in platelet function improvement are considered to be independent of weight loss or glycemic control in most studies [40,41,43,46] and are thought to be mediated by the reduction of inflammation and of insulin resistance [41,45]. Importantly, to date, no study has demonstrated a direct association between GLP-1 RA-induced modulation of platelet function and a reduction in thrombotic outcomes. Most available data derive from small, short-term studies evaluating surrogate platelet markers, and therefore, the clinical relevance of these findings remains to be established. Thus, more well-designed studies are crucial in this field. Furthermore, the available clinical evidence remains heterogeneous with respect to study populations (including individuals with T2DM, obesity, PCOS, and prediabetes), type and duration of intervention, and the platelet assays used. Therefore, the findings should not be interpreted as uniformly demonstrating a consistent antiplatelet effect across all clinical settings. While several studies suggest a potential improvement in platelet activation markers, differences in methodology and patient characteristics limit direct comparisons and preclude definitive conclusions concerning the generalizability of these effects.

**Table 2:** Clinical studies exploring the effects of GLP-RA treatment on platelet activation–related markers and indices.

Reference	Study Design	Population	Mean Age (Years ± SD)	GLP-1RA/Intervention	Platelet Function Outcome	Main Findings
Çalapkulu M et al., 2021 [40]	Single-center, case–control observational study	50 patients with T2DM with obesity (BMI > 35 kg/m^2^); 54 control subjects	Patients: 52 ± 9.2 Control group: 49.2 ± 5.4	Patients received exenatide treatment for six months	MPV; PDW; Platelet count	PDW and MPV were higher in the DM 2 group Positive correlation between PDW and BMI, FPG, and HbA1c; similar for MPV Significant decrease in PDW and platelet count after treatment
Kahal et al., 2015 [41]	Controlled interventional study ﻿	Obese women with a BMI between 30 and 45 kg/m^2^; 13 with PCOS versus 12 controls	Patients: 33.9 ± 6.7 Control group: 33.5 ± 7.1	Liraglutide for six months for both groups	P-selectin surface expression; fibrinogen binding; platelet response to activation by ADP or inhibition by PGI2	Significant inhibition of P-selectin expression in the control group, with no change in fibrinogen binding; no change in response to ADP or PGI2
Loganathan et al. 2022 [43]	Double-blind randomized controlled pilot trial	T2DM patients not receiving antiplatelet therapy and without macrovascular disease	Liraglutide group: 57.1 ± 5.7 Placebo group: 63 ± 6.7	Liraglutide treatment (1.8 mg/day) for 6 months	Platelet aggregation studies on platelet-rich plasma in the presence of an agonist (ADP, collagen, arachidonic acid, ristocetin, epinephrine)	Normal platelet aggregation in both groups; Significant, agonist-specific attenuation of the maximum slope of the aggregation response between days 0 (pre-treatment) and day 7 for liraglutide-treated patients compared to placebo
Simeone et al., 2018 [44]	Randomized controlled parallel-arm study	Obese patients (BMI > 30 kg/m^2^) with a diagnosis of IGT, IFG, or T2DM under diet therapy plus metformin treatment	Liraglutide group: 48–64 (mean: 55.5) Lifestyle intervention group: 51–55 (mean: 53)	For 6 months Liraglutide versus lifestyle interventions	Various markers and measurements as predictors of thromboxane-dependent platelet activation (SAT, VAT, HbA1c, Ln-TNF-α)	HbA1c, Ln-TNF-a and SAT (subcutaneous adipose tissue) were significant independent predictors of thromboxane-dependent platelet activation; lifestyle intervention and liraglutide therapy are equally effective to reduce the levels of U-11-dehydro TXB2, as well as U-8-iso-PGF2
Zhang et al., 2021 [45]	Observational case–control study	30 patients with newly diagnosed T2DM versus 30 healthy individuals	Patients: 40.23 ± 10.83 Controls: 43.10 ± 11.19	Patients received an 8-week exenatide treatment	NO, fibrinogen, CD62p, PAC-1, platelet aggregation induced by epinephrine, arachidonic acid, and ADP	Fibrinogen, CD62p, PAC-1, platelet aggregation lower than before treatment; NO level higher than before treatment.
Cahill et al., 2022 [46]	Interventional randomized clinical trial	Adults with obesity and prediabetes	40.3 ± 15.08 years	Patients were randomized in a 2:1:1 ratio to receive liraglutide, sitagliptin, or caloric restriction	Thromboxane-induced platelet aggregation: PRP was stimulated with U44619, and platelet aggregation was assessed at baseline and post-2-week treatment.	Two weeks of in vivo exposure to liraglutide reduced TX-induced platelet aggregation from baseline

Note: Clinical studies differ substantially in terms of patient populations, duration of exposure, type of GLP-1 RA used, and platelet assays performed. ADP: adenosine diphosphonate, BMI: body mass index, CD62p: P-selectin, FPG: fasting plasma glucose, HbA1c: hemoglobin A1C, Ln: natural logarithm, MPV: mean platelet volume, NO: nitric oxide, PDW: platelet distribution width, PGI2:prostaglandin-I2, PRP: platelet-rich plasma, PCOS: Polycystic ovary syndrome, PAC-1: platelet membrane glycoprotein IIb/IIIa complex, ROS: reactive oxygen species, SAT: subcutaneous adipose tissue, TNF-a: Tumor-Necrosis Factor A, T2DM: Type-2 Diabetes Mellitus, TX: Thromboxane, U-11-dehydroTXB2:Urine 11-dehydro-thromboxane B2, U44619: a synthetic analog of PGH2, U-8-iso-PGF2:8-iso-prostaglandin F2α, VAT: visceral adipose tissue.

## 6. Conclusions and Future Perspectives

Although GLP-1 RAs first emerged as glucose-lowering drugs, they have been established as first-line therapy in the context of metabolic and atherosclerotic disease owing to their cardiovascular protection, which has been proven in RCTs and verified from real-world data showing reductions in MACE associated with their use. Elucidating the mechanisms underlying their cardioprotective properties has been the subject of several experimental and clinical studies. These appear to extend beyond glycemic control and weight reduction and involve primary pathophysiological processes in atherothrombosis. While several studies have demonstrated beneficial effects of GLP-1 RAs on endothelial dysfunction, vascular inflammation, and oxidative stress, their impact on platelet function remains relatively understudied. Nevertheless, the pivotal role of platelet activation in the pathogenesis of atherothrombosis calls for a thorough understanding of the potential effects of GLP-1 RAs on platelet function. Importantly, platelet function is altered in both T2DM and obesity, the primary therapeutic targets for GLP-1 RAs [1,2,3,5,7,8,9,12,13,20,47].

Preclinical studies have shown that GLP-1 RAs attenuate platelet activation and thrombus formation through both GLP-1 receptor-dependent signaling and receptor-independent nitric oxide-mediated pathways, mainly under conditions of inflammation or shear stress. Small exploratory clinical studies suggest a potential reduction in platelet activation markers. However, these studies are limited by small sample sizes, short duration, heterogeneous platelet assays, and reliance on surrogate endpoints. Also, given the narrative, non-systematic design of this review, the findings should be interpreted with caution, particularly when extrapolating indirect mechanistic or clinical data.

While GLP-1 RAs may influence platelet-related pathways, clinical relevance and independence from weight loss or glycemic improvement remain uncertain. Large, appropriately designed studies are needed to verify the potential for modulating platelet function and signaling by GLP-RAs, which could affect therapeutic decisions for cardiovascular disease prevention and treatment in high-risk individuals.

Future research agendas in the field might include the following:

- Impact of treatment with GLP-1 RAs on megakaryocyte transcriptomics and epigenomics. Bulk and single-cell RNA sequencing can be applied to human megakaryocytes after in vivo GLP-1RA exposure to define GLP-1-dependent transcriptional and epigenetic signatures.
- Investigation of agent-specific effects by direct comparison of GLP-1 RAs with dual glucose-dependent insulinotropic polypeptide/GLP-1 agonists such as tirzepatide to determine differential effects on platelet activation and macrovascular outcomes.
- Better understanding of platelet–endothelium cross-talk. Future studies can explore how GLP-1 RAs modify platelet–endothelial interactions, including adhesion molecule expression and platelet-derived inflammatory mediators.
- Effect of GLP-1 RAs on microvascular function: combination of platelet assays with established non-interventional markers of microcirculation to link platelet modulation with microvascular protection. Similar studies on other clinical entities can be inspirational for this aim [48,49,50,51].
- Development of precision medicine approaches. We can identify clinical or molecular phenotypes predicting platelet responsiveness to incretin-based therapies. Collaboration with machine learning and artificial intelligence scientists is crucial in this field.

## Data Availability

No new data were created or analyzed in this study. Data sharing is not applicable.

## Abbreviations

The following abbreviations are used in this manuscript:

ADP	Adenosine diphosphate
AERD	Aspirin exacerbated respiratory disease
BMI	Body mass index
cAMP	Cyclic adenosine monophosphate
cGMP	cyclic guanosine monophosphate
CRP	C-reactive protein
CT	Computed tomography
DPP-4	Dipeptidyl peptidase 4
eNOS	Endothelial NO synthase
FMD	Flow-mediated dilation
FPG	Fasting plasma glucose
GLP-1	Glucagon-like peptide-1
GLP-1R	Glucagon-like peptide-1 receptor
GLP-1RA	Glucagon-like peptide-1 receptor agonist
HbA1c	Hemoglobin A1c
Hs-CRP	High-sensitivity C-reactive protein
IL	Interleukin
LDL	Low-density lipoprotein
MACE	Major cardiovascular events
MCP-1	Monocyte chemoattractant protein-1
MPV	Mean platelet volume
NO	Nitric oxide
PCOS	Polycystic ovary syndrome
PDW	Platelet distribution width
PG	Prostaglandin
PKA	Protein kinase A
RCT	Randomized controlled trial
SUVmax	Maximum standardized uptake value
T2DM	Type 2 diabetes mellitus
